# Photon Parameterisation for Robust Relaxation Constraints

**DOI:** 10.1111/cgf.12028

**Published:** 2013-05-07

**Authors:** B Spencer, M W Jones

**Affiliations:** Visual and Interactive Computing Group, Swansea UniversityUK

## Abstract

This paper presents a novel approach to detecting and preserving fine illumination structure within photon maps. Data derived from each photon's primal trajectory is encoded and used to build a high-dimensional kd-tree. Incorporation of these new parameters allows for precise differentiation between intersecting ray envelopes, thus minimizing detail degradation when combined with photon relaxation. We demonstrate how parameter-aware querying is beneficial in both detecting and removing noise. We also propose a more robust structure descriptor based on principal components analysis that better identifies anisotropic detail at the sub-kernel level. We illustrate the effectiveness of our approach in several example scenes and show significant improvements when rendering complex caustics compared to previous methods.

## 1. Introduction

Caustics, or the family of light transport paths that contain a *S*^+^*D* sub-path, are responsible for some of the most complex and distinctive visual phenomena found in nature. As a component of the rendering equation, however, they often prove challenging to solve owing to the relatively tiny proportion which carry a significant contribution of energy between the emitter and the eye. Consequently, caustics require the use of bi-directional and multi-pass rendering techniques which simulate energy flow both forwards from the light source and backwards from the eye. Among the most popular of these is photon mapping [Jen96b]: a two-pass approach that has seen widespread adoption thanks to its speed, robustness and extensibility.

A typical photon map stores packets of radiant flux at diffuse surfaces which have first undergone specular reflection and refraction. The exitant radiance from the caustic component at any given point can be reconstructed from the photon distribution using a density estimation kernel. This approach capitalises on the correlation between the integral of incident illumination at each point on a surface, transforming the sparse photon point set into a continuous function of illumination. The principal drawback of the photon mapping method is the relatively low fidelity of the cached dataset which generally results in error being introduced into the radiance reconstruction.

Error reduction strategies for density estimation algorithms have been rigorously researched and a large body of work now exists which addresses nuances of the problem specific to the photon mapping framework. Photon relaxation [SJ09] represents a recent contribution that aims to reduce error by directly diffusing the underlying point distribution. Though shown to be effective at smoothing out multi-frequency noise, this approach can significantly degrade intricate, subtle or high frequency detail.

After covering the background literature in Section 2 and introducing the problem in Section 3, this paper:

- Discusses the advantages of augmenting the standard photon map with information about each photon's initial trajectory. After motivation in Section 4, the chosen parameters are proposed in Section 4.1.
- Explores the new k-NN query enabled by this approach (Section 4.2) along with the idea that the photon kd-tree can be extended to a higher-dimensional space thereby allowing efficient, parameter-aware querying. These combine to effectively isolate overlapping or interfering illumination.
- Introduces a new method of identifying anisotropic structure within the photon distribution using principal components analysis (Section 4.3).
- Presents and discusses the resulting denoised images in Section 5. Due to the low kernel bandwidths made practical by this approach, low render times and low bias are achievable whilst still yielding low noise images.

## 2. Background

Error in the radiance reconstruction from the photon map is the sum of two constituent components: noise and bias [Sil86]. Noise appears as a speckling artefact arising from discrepancy within the underlying point distribution coupled with variance in the stored flux between photons. The radiance estimator acts as a filter for noise, the upper frequency of which is bounded by the bandwidth and support of the kernel. Naïve estimators adapt poorly to changes in the flux density function and result in undesirable smoothing known as bias. These conditions result in a trade-off where a decrease in noise yields an increase in bias and vice versa. Despite this correlation, bias and noise are only weakly codependent. This implies that a well-designed kernel can adapt to the underlying density function, reducing noise while minimising the penalties from bias.

Jensen and Christensen [JC95] were the first to address intelligent bandwidth selection within the photon mapping framework with the introduction of differential checking. Later, Schregle [Sch03] defined a bias compensation operator using the statistical properties of random numbers to differentiate between noise and bias. Hey and Purgathofer [HP02] proposed utilising data from the underlying polygonal mesh to address the twin problems of boundary and topological bias.

Havran et al. [HBHS05] demonstrated how storing the photon ray paths could be used to more accurately estimate radiance on intricate geometry. Herzog et al. [HHK*07] sought a solution by splatting photons directly onto eye samples projected from the image plane. Chen et al. [CTC10] used a clustering strategy to group photons with coherent ray paths into small, individual photon maps analogous to light beams.

Progressive photon mapping [HOJ08] solves the problem of bias by reducing the kernel radii of a series of pre-cached estimates as energy from successive passes of incident photons is accumulated. Given sufficient time, the bias of each estimate converges asymptotically to zero thus making the algorithm consistent in the limit [HJ09]. Knaus and Zwicker [KZ11] proposed a memoryless adaptation of progressive photon mapping which also extended the algorithm to support arbitrary kernels and participating media.

More diverse methods of bias reduction have also been developed for the kernel density estimation framework [SWH*95]. Myszkowski [Mys97] proposed calculating the error due to bias from an array of neighbouring estimates adjacent to the sample positions. Walter et al. [WHSG97] addressed the problem with a solution based on polynomial regression and augmented with a perceptually-driven bandwidth selector [Wal98] to account for variance both in luminosity and chromaticity.

Schjøth et al. [SOS05] demonstrated the limitations of isotropic, variable bandwidth kernels and proposed that filtering the estimate anisotropically could dramatically improve results. Later, Schjøth et al. [SFES07] adapted the ray differential framework [Ige99] to shape the filter based on photon ray footprints. Jakob et al. [JRJ11] used a hierarchically accelerated expectation-maximisation algorithm to decompose the photon map into a series of anisotropic Gaussians, thus allowing more efficient rendering of participating media.

Photon relaxation [SJ09] addresses the problem of noise by directly redistributing the underlying point distribution via an intermediate pass between particle tracing and rendering. Using iterative point repulsion, noise is diffused away leaving a locally equidistributed point set with a blue noise spectral signature. Research has shown that blue noise distributions are optimal in many sampling applications [Uli88] and their low-noise properties have also been found to apply to the photon map. The relaxed photon map is of a sufficient fidelity to allow very low bandwidth radiance estimates. This has the effect of minimising topology, proximity and boundary bias and reduces render time. Spencer and Jones later developed a progressive approach to photon relaxation [SJ13] by using successive passes of temporarily stored photons to refine an estimate of flux density over the Voronoi cells of a conventional photon map. A capacity constraint equation is used to iteratively refine cell areas yielding an accurate estimate of per-photon flux density encoded by an optimised blue noise distribution.

## 3. Problem Definition

Photon relaxation as defined by Spencer and Jones [SJ09] uses iterative point repulsion to progressively remove noise from a photon distribution. Though effective, the process of compound diffusion can also act negatively to degrade the encoded flux density function, *B*. By selectively constraining photon migration, the undesirable effects of diffusion can be counteracted, preserving important visual structure while still effectively removing noise.

In determining the degree of constraint to apply to an individual photon, we refer to general models of diffusion for a correspondence. In particular, Fick's first law states that the flux of a diffusive system is proportional to the gradient of the density [Fic55]. In the context of photon relaxation, this is analogous to the rate of migration of photons across a surface. Hence, photons should be constrained in the direction of and proportional to the magnitude of the gradient, ∇*B*, measured from the flux density function.

**Figure 2:**
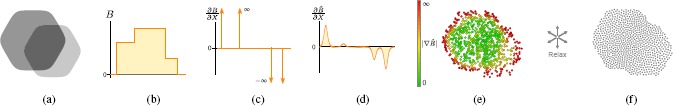
The discontinuity problem. (a) A sample PDF (inverted for clarity) of flux density, B. (b) Plotting B along a slice through the PDF reveals its discontinuous structure. (c) An analytical differential is analogous to a series of delta functions at the discontinuities. Here, the magnitude of the gradient is infinite indicating the need to maximally constrain photons at these points. (d) The estimate of B reconstructed from the photon distribution is biased, yielding finite values of ∂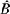 proportional to the change in flux density. These estimates do not reliably indicate discontinuities. (e – f) Mapping |▿
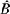
| to photon constraints and relaxing the distribution results in the interior structure of the PDF becoming severely degraded.

Since *B* and by extension ∇*B* are unknown, they must be reconstructed from the photon distribution. It is this process of estimation that introduces an intrinsic limitation in the photon relaxation algorithm and which our new approach attempts to address. To demonstrate the problem, we sketch a sample PDF with a piecewise, discontinuous profile and plot it in Figures 2a and b. Analytically differentiating to obtain an exact value of ∂*B* yields the plot in Figure 2c. Note how the infinite gradient on each discontinuity yields a series of delta functions with antiderivatives equal to the absolute change in flux density. Photons at these locations will lie on a transition at the highest possible spatial frequency and should therefore be maximally constrained.

In the absence of an analytical derivative of *B*, the estimate 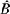 is used instead. This must be derived from the photon distribution local to the query point and is therefore intrinsically biased. Estimator bias effectively acts to convolve or smooth the flux density function. We depict this effect in Figure 2d where the energy at the singularity of each delta function has been redistributed along the spatial axis by the kernel. This serves to weaken the estimated gradient which is now always finite. Critically, the estimate is also ambiguous since there is no way of separating the bias from the original function. For example, it is impossible to determine whether a weak gradient estimate is the product of a relatively shallow yet still discontinuous transition that has been smoothed by the kernel, or else a legitimately weak slope of *B*.

**Figure 1:**
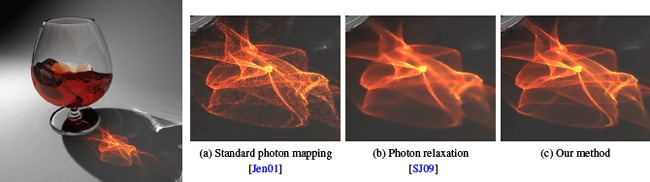
Cognac Glass. (a) An unmodified photon map rendered with 15 photons in the radiance estimate exhibits high levels of noise. (b) Photon relaxation as proposed by Spencer and Jones virtually eliminates noise, however overlapping filaments of the caustic are also degraded. (c) Our approach separates photons in a parametrised domain, correctly inhibiting migration even across interlaced boundaries.

The impact of this effect is demonstrated in Figure 2e where a photon distribution seeded using the PDF in Figure 2a is constrained according the biased gradient estimate. Notice that weak or non-existent constraints on interior boundaries lead to degradation of the density function after relaxation (Figure 2f). A practical example of this phenomenon appears in Figure 1 where photons are propagated through a model of a glass containing agitated liquid. In Figure 1a we render the unmodified photon map using 15-nearest neighbours in the radiance estimate. Using a low-bandwidth kernel maximises the fidelity and detail of the caustic but introduces noise as a result of the discrepancy in the distribution.

In Figure 1b the photon map has been diffused by 14 iterations of photon relaxation [SJ09]. Although this technique preserves sharp caustic boundaries, overlapping filaments of the caustic are blurred excessively due to the diffusion of photons. We note that this can be superficially addressed by turning up the gain on the gradient detection. Amplifying the constraints around shallow discontinuities curbs the effects seen in the Figure 1b but also amplifies error due to noise. This is a significant problem since photon maps are inherently noisy. Overzealous constraint in otherwise uniform regions of illumination introduces an undesirable contouring effect, examples of which can be seen in [SJ09]. Furthermore, the user-specified parameters required to optimise gain for individual photon maps makes this approach difficult to control.

**Figure 3:**
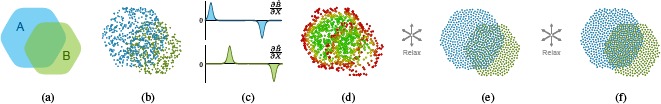
(a – b) Our method seeks to address the discontinuity problem by storing additional information at each photon – in this case whether it belong to set A or B. (c) This effectively separates gradient estimation into two different domains for each set. (d) When applied to the photon map, we can see that the boundary of each set is now properly constrained. (e) Parameter-aware relaxation rapidly removes noise but generates a sub-optimal distribution where the sets overlap. (f) Collapsing the extra dimensions and relaxing again results in the correct blue noise signature.

What is needed is a means by which to treat all discontinuities with equal salience, thus allowing appropriate migration constraints even when the biased gradient estimate is unreliable. Most importantly, the system must be resilient to noise in order to avoid rendering artefacts. To accomplish this, we propose separating features within the photon distribution within a new, parametrised domain so as to make feature detection more resilient to kernel bias. This concept is illustrated in Figure 3b where the PDF from Figure 2a has been broken up into two individual sets. Feature detection on these sets individually yields more consistent gradient estimates and allows more robust photon constraints. In the following section, we explore a practical method of parameterising the photon map so that caustic illumination may be dissociated in the same fashion.

## 4. Our Method – High-Dimensional Parameterisation

In order to devise a more robust method by which to break down and separate the complex visual structure found in many caustics, it is useful to consider the physical model behind their formation. This allows us to define new parameters which can be used to differentiate between distinct structural elements like those found in the cognac caustic in Figure 1.

**Figure 4:**
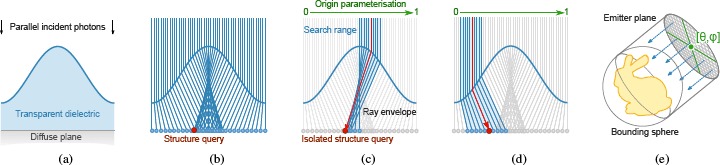
(a) Light passes from a vacuum into a transparent dielectric before being diffusely reflected. (b) Photons are focussed toward the centre of the ground plane producing a prominent discontinuity in flux density at the point marked in red. Querying the gradient of the photon map at this point results in photons outside of the envelope also being included, weakening the boundary constraint. (c) Parameterising the incident ray origin means we can restrict which photons are included in the gradient estimate to those that are local to the queried photon in parameter space, thus preventing non-local photons from interfering with the structure extrapolation. (d) Furthermore, photons lying near but not on features are not affected. (e) Collimated emitters use the same parameterisation but over the 2-dimensional space of photon origins, [θ,ϕ].

A discontinuity within a caustic may be formed in one of two ways. The first is through occlusion where one piece of geometry physically obscures another. Photons are captured by the occluding surface thereby creating a shadow on any surface beneath it. The second type of discontinuity appears as the result of an intersection between a photon ray envelope and a diffuse surface. This concept is demonstrated in Figure 4b where collimated light from an overhead source is refracted by a transparent dielectric interface in the shape of a wave. Two ray envelopes are visible to the left and to the right which together form an inverted, V-shaped curve directly below the apex of the dielectric. The discontinuity in photon density defines the locus where each ray envelope intersects the surface. In order to prevent migration across these features during relaxation, photons should be maximally constrained along each boundary.

We introduce our method with the observation that specular caustics represent a warping of the primal distribution by intermediate geometry. Groups of photons that are sequential at the point of emission may subsequently become folded together as they are reflected and refracted. By isolating and grouping stored photons based upon a parameterisation of their initial trajectories, we can effectively unfold complex, overlapped caustic elements in a meaningful way. Specifically, interference from non-local photons can be excluded while still preserving discontinuities which arise from occlusion and ray envelopes. This facilitates more effective edge detection since only the region of the distribution parametrically local to the photon being queried is considered for feature extrapolation.

**Algorithm 1:**
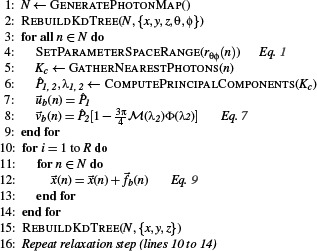
PhotonRelaxation()

In Figures 4c and 4d, the incident rays have been mapped to a one-dimensional parameterisation in the range [0,1 based upon their origin. The value of this parameter is subsequently encoded at each stored photon. Computing the gradient at the photon marked in red requires that we not only search for photons local in world space, but also in parameter space (highlighted in blue). When the path of the queried photon lies on the ray envelope, parameter localisation has the effect of excluding those photons that are not part of its structure. Furthermore, when a photon from a low density region is queried, proximity bias from the adjacent high density region does not affect the gradient estimate (Figure 4d).

In the next section we discuss suitable parameterisations for common light sources that will enable us to appropriately separate distinct caustic filaments.

### 4.1. Photon Emitter Parameterisations

The parameterisation of incident illumination in Figure 4 is derived from the relative origin of each photon and can be extended to two dimensions to handle illumination from collimated light sources such as the sun. This is illustrated in Figure 4e where photons are cast from an emitter plane which has been parametrised into two orthogonal axes. We designate these parameters θ and ϕ.

**Figure 5:**
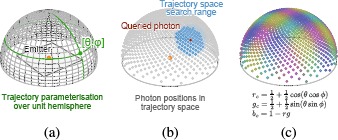
Parameter space constraints. (a) For a Lambertian point emitter, the primal photon trajectory over the unit hemisphere yields a pair of spherical coordinates, [θ, ϕ]. (b) During feature detection, k-NN querying is restricted to a fixed range in parameter space (Equation 1) which aids in separating individual caustic filaments. (c) The colour encoding used to visualise photon parameter space positions in Figures 6e and 8d.

For emitters at a finite distance we are forced to use a different mapping since the origin of each photon path is likely to be relatively invariable. We observe that the change in position of absorption of photons from a point source is a function of the exitant angle of emission which can summarily be mapped onto the spherical coordinates of the unit space around the light source. This is depicted in Figure 5a where the unit hemisphere above a Lambertian point emitter has been parametrised based upon the inclination and azimuth angles. Omni-directional emitters can use the same parameterisation based on the unit sphere.

Photons stored in the photon map are encoded with the (*x, y, z*) tuple of their spatial position together with their coordinates in parameter space. For collimated emitters this adds two extra dimensions. For Lambertian and omni-directional emitters it adds three since their 2-dimensional spherical coordinates must be embedded in 3-dimensional space. In Algorithm 1, kd-tree construction is encapsulated by the function RebuildKdTree(N,{…}). The first parameter, *N*, is the set of all photons. The second parameter indicates the set of dimensions partitioned by the tree; {*x,y,z*} for world space position and {θ,ϕ} for emitter parameterisation.

### 4.2. Feature Detection and Relaxation

K-nearest neighbour searching is performed for the lower three Euclidean dimensions while constrained to a range in parameter space (Figure 5b). The extent of the neighbourhood in parameter space within which to search determines the effectiveness at isolating intersecting ray envelopes. Too large a radius and overlapping regions of the caustic may not be correctly differentiated. Too small and feature detection may incorrectly interpret warping between parameter and world spaces as structure.

We determine the search radius on a per-photon basis from the local photon density, ρ. We define ρ as the number of photons per unit area of the 2-manifold parameter space of the emitter which we measure *a priori* to feature detection. Hence, for a given photon, *i*, its search range in parameter space is equal to:


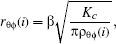
(1)

where *K_c_* is the number of nearest neighbours used to determine the structure of the photon distribution. The scale factor, β, allows vital flexibility to account for warping and distortion in the mapping between parameter and world spaces. In our implementation, we found a value of **β**= 3 consistently differentiated between ray envelopes while avoiding incorrect constraint.

We perform feature detection at every photon by gathering its *K_c_*-nearest neighbours in world space and constraining the search within radius *r*_θϕ_ to the parameter space coordinate of the photon being analysed (illustrated by Figure 5b and encapsulated by SetParameterSpaceRange() and GatherNearestPhotons() in Algorithm 1). An orthogonal basis, *b*, is then computed into which the force of repulsion at every iteration is projected. We explore this process in detail in the following section.

### 4.3. An Improved Anisotropic Structure Descriptor

**Figure 6:**
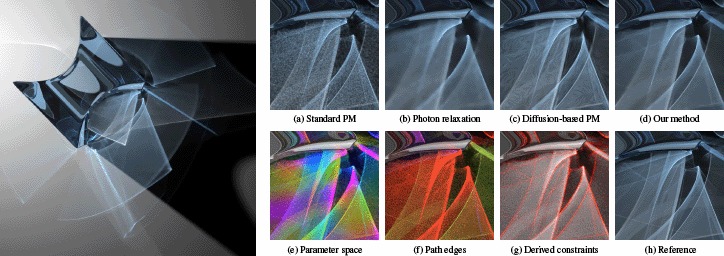
A glass prism illuminated by a strong point light. Caustic illumination is encoded by approximately 880,000 photons. 15-nearest neighbours were used in the radiance estimate except in the case of (c) where the minimum diameter of the anisotropic kernel is adjusted to yield comparable levels of bias.

A second effect of the bias introduced by the gradient estimator is unpredictable behavior on or near structures that are both anisotropic and radially symmetric within the span of the kernel. Prominent examples can be seen in Figure 6a where the distribution exhibits fine filaments only one or two photons wide. Basing the gradient estimate, ∇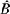, on the density derivative fails to detect these structures because the relative mean cancels to zero in all directions (Figure 6b). Significantly, the problem is not addressed by our high-dimensional parameterisation because the structures we are trying to detect exist at a frequency above that of the kernel used to estimate them.

#### 4.3.1. Principal Components Analysis

As a solution we propose a more robust structure descriptor based upon principal components analysis [Pea01]. PCA is useful because the transformation defines both a scalar measure and dominant axis of anisotropy. Most importantly, it is not rendered ineffective by radially-symmetric distributions.

The principal components for the k-nearest neighbors local to some photon, *i*, are found by computing the eigendecomposition of the covariance matrix derived from the zero-mean point set. To reduce the dimensionality and subsequent complexity of the decomposition, we project each nearest neighbor into 2-dimensional tangent space derived from the surface normal of the photon for which we are computing the constraint. The resulting pair of eigenvectors, 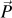
_1,2_, form an orthogonal basis into which the photon's repulsion vector is projected during relaxation. The corresponding eigenvalues, λ_1,2_, indicate the degree of anisotropy of the distribution from which we compute the level of constraint. Throughout the text, values of λ are normalised to the radius of the kernel, *r*. This entire process is encapsulated by ComputePrincipalComponents() in Algorithm 1.

Regions where the photon distribution is highly isotropic indicate low variation in incident illumination. Under these conditions, photons can migrate freely as required by the relaxation function. Increasing anisotropy corresponds to an increasingly steep gradient. The magnitude of the eigenvalue of the second principal component, λ2, indicates the degree of anisotropy and can be used to specify the level of constraint along the axis defined by its corresponding eigenvector.

We aim to limit the motion of photons in the gradient direction so that the degree of constraint is proportional to the pressure exerted upon them by their nearest neighbors. We first restate that the migration pressure applied to each photon is proportional to the density derivative, ∇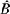. To this end, we establish a bijective mapping, ℳ, between λ_2_ and |∇
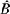
| of the distribution within the k-NN kernel around photon *i*:


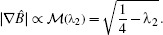
(2)

**Figure 7:**
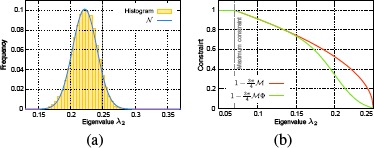
(a) The histogram of samples of λ2 measured from 100,000 unique sets of an isotropic test distribution. (b) The plot ofλ_2_ against the magnitude of the resulting constraint computed by our mapping function. In both frames, K_c_ is set to 100.

Equation 2 is plotted in red in Figure 7b over the range [0, 0.25]. The limit marked “maximum constraint” corresponds to the migration pressure on a photon on the edge of a maximal discontinuity. At this point, in order to prevent blurring, no movement in the gradient direction should be permissible. A full proof of the derivation of Equation 2 is given in section A of the supplementary material.

#### 4.3.2. Filtering Noise

We also consider the signal noise floor and its effect on feature detection. Photon maps are intrinsically noisy and artefacts may be introduced if the algorithm misidentifies noise as structure. To ensure robust noise removal while preserving important visual features, we propose a statistical method of filtering erroneous results.

We first acknowledge that the measure of anisotropy defined by λ_2_ is a function of both the background noise in the local photon distribution and the underlying flux density function. Since we do not know either with exact certainty, these components are not explicitly separable. However, we can draw upon certain properties of random numbers to infer a statistical likelihood that a detected feature is merely a result of noise and not a change in flux density.

The central limit theorem states that the sum of a set, ξ, of *K* independent and identically distributed random variables will be normally distributed. The usefulness of this rule lies in the fact that each variable may have an arbitrary PDF. Thus the eigenvalues of the PCA decomposition of photons seeded using independent and identically distributed random numbers should also exhibit the same distribution. We confirm this experimentally by introducing a test case whereby a set of photons of size *K_c_* are seeded within a unit circle according to a continuous uniform distribution. In Figure 7a we plot the histogram of λ_2_ measured from 100,000 unique sets of our test case, each containing 100 photons. The mean of these data is measured to be approximately 0.2219 and the standard deviation 1.88 × 10^−2^. We overlay a Gaussian function, 𝒩, with equal mean and variance and see that they correspond with only a marginal degree of error.

We can use the properties of the central limit theorem to determine the attributes of the normal distribution for arbitrary values of *K_c_* in the feature detection kernel. The probability of the sum of random variables, Σξ, being less than real value, *x*, converges to the CDF, Φ, of a standard normal random variable:


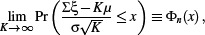
(3)

where μ and σ are the mean and standard deviation respectively. Crucially, this shows us that as *K* grows large, the magnitude of σ relative to μ diminishes asymptotically according the reciprocal of 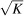. Since σ_𝒩_ is measured as 1.88 × 10^−2^ when *K_c_*= 100, the general equation for standard deviation becomes:


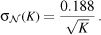
(4)

The equation for the mean, μ_𝒩_, can be computed using a similar relationship. Figure 7a demonstrates that *E*(λ_2_) when *K_c_* < ∞ does not equal λ_2_ as *K_c_*→∞. This is due to the interdependence of the two principal components which, by definition, require that λ_2_ be no greater than λ_1_. We can prove analytically that λ_2_= 0.25 when *K_c_* is infinitely large (section A in the supplementary material). Applying the same principal as Equation 4 and observing that λ2 converges asymptotically to 0.25, we express μ_𝒩_ as:


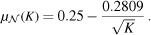
(5)

We restate our goal of attenuating the constraint applied to a photon according to the likelihood that the estimate of local anisotropy has been affected by noise. To achieve this, we compute one minus the integral of 𝒩 between –∞ and λ2. This is equal to the probability, Φ, that λ2 is less than or equal to the second eigenvector from a random sample set from the isotropic test case. The corollary is the estimated likelihood that the value of λ_2_ is not attributable to noise. This integral is defined as:


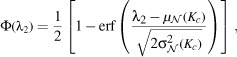
(6)

where erf is the error function. Equation 6 can now be applied as a continuous attenuation function that we use to de-constrain photons according to the detected degree of noisiness. We elaborate on this concept in the following section.

#### 4.3.3. Defining a Constraining Basis

Thus far we have derived a continuous correspondence between the force acting on a given photon and its PCA eigenvalues. From this we can derive its constraint as the *u* and *v* components of orthogonal basis, *b*:


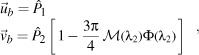
(7)

where 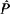 are the unit eigenvectors of the first and second principal components. Equation 7 is derived from Equation 2, adjusted for noise by Equation 6. Note that to prevent migration constraints outside the range [0,1], the computed value of λ_2_ must be clamped to 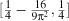 (see section A in the supplementary material for details). Given that the principal components, 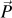
_1,2_ are computed in 2-dimensional photon tangent space, 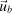 and 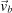 must be re-embedded into world space before being stored in the photon.

In Figure 7 we plot the migration constraints derived from Equation 7. The red curve effectively represents the unfiltered mapping, ℳ, from Equation 2. Applying noise compensation, Φ, attenuates the constraint as λ2 approaches its isotropic limit of 0.25 (green curve).

### 4.4. Relaxation

Once orthogonal bases have been found for every photon, the photon map is iteratively relaxed. We define the force, 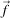, applied to every photon *i* at each iteration as:


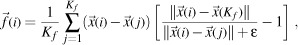
(8)

where *x* is the position of *i* in world space, ε is a arbitrarily small constant and *K_f_* is the set of nearest neighbours to *i* which we set to a value to 16. 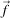 is summarily projected into basis *b* to enforce migration constraint:


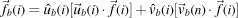
(9)

where û and 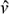 are the normalised basis vectors.

In this step, we take further advantage of the high-dimensional kd-tree by breaking the process down into two separate stages. The first stage relaxes the distribution by gathering photons within the high-dimensional constraints used by the feature detection algorithm. In the second stage, all dimensions extraneous to world space are summarily collapsed and the kd-tree rebuilt. Iterative relaxation is then repeated.

We demonstrate this progression in Figure 3e–f. We observe that the rate of noise diffusion is proportional to the relative density of the photon distribution. Hence, by relaxing the two sets independently, noise is diffused more uniformly than if they are treated as a single group. The drawback of this approach is that the desirable property of photon equidistribution is not attained where two or more sets intersect (as evident in frame d). To address this, we collapse the tree so the relaxation operator can no longer make the distinction between photon positions in parameter space. Because the component distributions, A and B, are already denoised, only a small number of iterations are required to yield an optimised point set (frame e).

## 5. Results

We compare our algorithm against the classic photon mapping algorithm introduced by Jensen, the original photon relaxation method [SJ09], diffusion-based photon mapping [SOS08] and, for reference, progressive photon mapping [HOJ08]. All tests were performed using an Intel Core i7 with 8GB of RAM running Windows 7. Both feature detection and relaxation were well-suited to vectorisation so we were able to utilise a total of 7 cores running in parallel. Timings for Figure 6 are listed in Table I (see section B in the supplementary material for further timings).

**Table 1:** Render timings for the close-up of the Prism scene (Figure 6) for the various demonstrated techniques. Image resolution is 600 × 600 with 4× supersampling.

	Standard PM	Photon Relax.	Diff-Based PM	Our method	Progressive PM
*M*/ Absrbd. *M*	20M / 863k	20M / 863k	20M / 863k	20M / 863k	4.6B / 200M
Photon cast *t*	12.3s	12.3s	12.3s	12.3s	47m 21s
Radiance *K*	15	15	500	15	Variable
Feature detect *t*	–	9.1s	3.3s	5.3s	–
Relaxation *t*	–	27.3s	–	28.4s	–
Render *t*	4.3s	4.3s	1m 29s	4.3s	–
Total *t*	14.8s	48.2s	1m 44s	50.3s	47m 21s

With the exception of the reference images, exitant radiance was reconstructed using an Epanechnikov kernel filter [Sil86]. When casting photons, the domain of each emitter was sampled using a low discrepancy, quasi-random Halton sequence. We use a value of *K_c_*= 50 in the feature detection kernel from which are derived the principal components. For our method, the number of relaxation iterations (parameter *R* in Algorithm 1) is set at 7 in high-dimensional space followed by a further 7 in collapsed world space. For the original photon relaxation method, 14 iterations were performed in collapsed world space.

**Figure 8:**
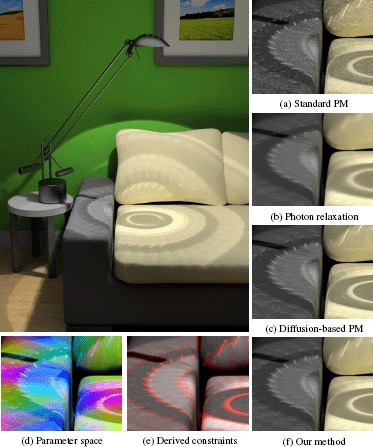
A halogen reading lamp illuminating a chair. All illumination in this scene is caustic since it has first passed through a glass shield covering the bulb. The intricate patterns are caused by a faceted parabolic reflector mounted above the bulb.

When rendering with diffusion-based photon mapping, we used 500-nearest neighbours to achieve comparable levels of noise removal. Schjøth et al. [SOS08] specify a diffusivity function [PM90] which controls the anisotropy of the kernel filter. To yield visually comparable levels of blurring due to kernel bias we set the control parameter, *q*, to 0.02. This yields similarly sharp edges around discontinuities in illumination, although it also introduces artefacts from noise (Figures 6c and 8c). Increasing *q* alleviates these effects at the expense of increased smoothing on overlapping caustic filaments.

Figure 1 is based on Jensen's classic Cognac glass [Jen96a] which has been shaken to agitate the liquid within it. This scene highlights the advantages of high-dimensional feature detection over its low-dimensional counterpart. Our method successfully constrains photons on weak discontinuities unlike the previous method which allows them to diffuse away.

In Figure 6, a glass prism generates intricate shards of caustic illumination when lit from behind. In frame c we use the anisotropic, shape-adapting kernel introduced by Schjøth et al. [SOS08]. The visible contouring effect is due to noise being misidentified as structure. Frame d has been denoised by our method. Note how caustic filaments are well-preserved, even those that are visually difficult to distinguish in frame a. Frames e and f are rendered using colour encodings of photon parameter space and path length. In frame g, highly constrained photons are highlighted in red. Finally, frame h depicts a reference image rendered with progressive photon mapping.

Figure 8 depicts a halogen reading lamp illuminating a chair. The complex pattern is caused by the faceted parabolic reflector above the bulb mounted behind a glass filter. The subtle faceting is barely represented by the noisy photon map (Figure 8a), however our trajectory space parameterisation (Figure 8d) successfully separates and preserves its internal structure (Figure 8f).

**Figure 9:**
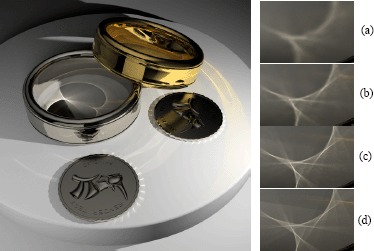
Rings and Coins Redux. Close-up on our method using (a) 134k, (b) 670k and (c) 3.3M photons. (d) Rendered with PPM using 1B integrated photons. Wide shot was rendered using the photon map from frame b.

Finally, Figure 9 demonstrates the performance of our approach using variable numbers of photons.

## 6. Conclusion

In this paper we have demonstrated how a high-dimensional kd-tree built using data parametrised from each photon's primal trajectory is more effective at differentiating between complex visual structure that naturally occurs in caustic illumination. We have also introduced a more robust approach to feature detection that correctly handles anisotropic distributions and is tolerant to noise. Our method has been shown to be effective at removing noise while preserving important visual detail.

As future work we would like to explore other potential parameterisations besides those of the light source, for example, angle of photon incidence, path length and number of edges (Figure 6f). Also of interest is the ray differentials framework as adapted to photon mapping by Igehy [Ige99]. We would also like to extend our technique to work with volume photon maps and investigate temporally coherent photon relaxation for applications in animation.
