# Supplementary material for: Photon Parameterisation for Robust Relaxation Constraints
Source: Comput Graph Forum. 2013 May 7;32(2 Pt 1):83–92. doi: 10.1111/cgf.12028 (PMC3744758; doi:10.1111/cgf.12028)
Supplement: Supplementary file 1 [file cgf0032-0083-sd1.pdf]

Photon Parameterisation for Robust Relaxation Constraints

Supplementary Material

Ben Spencer and Mark W. Jones
Visual and Interactive Computing Group, Swansea University, UK

A Bijective Mapping, $\mathcal{M}$, Between λ_2 and $|\nabla \hat{B}|$

We derive an analytical correspondence between the magnitude of the gradient estimate, $|\nabla \hat{B}|$, and the second eigenvalue, λ_2, of the PCA transform over the subset of nearest neighbors, K, to point $\vec{x}$. We first acknowledge that the photons in K encode a product of the incident flux density function, B, and the circular density estimation kernel. We establish that the density derivative, $\nabla \hat{B}$, is analogous to the empirical mean of this product. Furthermore, our chosen indicator of isotropy, λ_2, is equivalent to the variance of the product in the plane defined by $\vec{P}_2$. Since the variance of a function is analytically derived from its mean, we aim to find an inverse that expresses mean in terms of variance and, by extension, migration pressure.
The space of all possible flux density products is complex necessitating a simplified case from which to derive a bijective correspondence. We use as a model a unit radius kernel, g, that exhibits a discontinuous density function, controlled by a single parameter, a. We illustrate some examples in Figure 1 (bottom) for varying values of a and also demonstrate a sample distribution using 1000 points in the top of that figure.

$$g(u,v) = \begin{cases} a, & \text{when } -1 < u < 0 \text{ and } u^2 + v^2 \leq 1 \\ 1-a, & \text{when } 0 \leq u < 1 \text{ and } u^2 + v^2 \leq 1 \\ 0, & \text{otherwise} \end{cases} \tag{1}$$

We integrate $g(u, v)$ over dv and normalize by kernel area to give a probability density function dependent only on u.

$$g(u) = \int_{-\infty}^{\infty} g(u,v)dv = \frac{4}{\pi} \begin{cases} a\sqrt{1-u^2}, & \text{when } -1 < u < 0 \\ (1-a)\sqrt{1-u^2}, & \text{when } 0 \leq u < 1 \\ 0, & \text{otherwise} \end{cases} \tag{2}$$

The mean value, μ, of g is defined as:

$$\mu_u(g) = \int u\, g(u)\, du \tag{3}$$

which expands to:

$$\begin{aligned} \mu_u(g) &= \frac{4a}{\pi} \int_{-1}^{0} u\sqrt{1-u^2}du + \frac{4(1-a)}{\pi} \int_{0}^{1} u\sqrt{1-u^2}du \\ &= \frac{4}{3\pi}(1-2a), \end{aligned} \tag{4}$$

In turn, the variance, Var_u is defined as:

$$\text{Var}_u(g) = \int (u - \mu(g))^2 g(u) du, \tag{5}$$

which expands to:

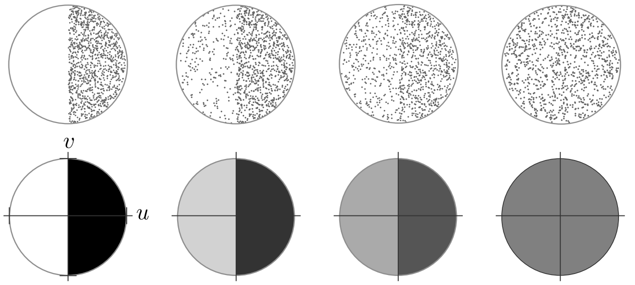

Figure 1: The gradient function for various values of a.

$$\begin{aligned} \mathrm{Var}_u\,(g) &= \frac{4a}{\pi}\int_{-1}^{0}(u-\mu(g))^2\sqrt{1-u^2}\,du \\ &\quad + \frac{4(1-a)}{\pi}\int_{0}^{1}(u-\mu(g))^2\sqrt{1-u^2}du \\ &= \mu(g)^2 + \frac{8\mu(g)}{3\pi}(2a-1) + \frac{1}{4} \end{aligned} \tag{6}$$

Substituting for a and solving yields:

$$\mathrm{Var}_u\,(g) = \frac{1}{4} - \mu^2 \tag{7}$$

For any value of a in the interval $[0, 0.5]$, $\mathrm{Cov}_{u,v}\,(g) = 0$, $\mathrm{Cov}_{v,u}\,(g) = 0$ and $\mathrm{Var}_v\,(g) = \frac{1}{4}$. Hence, using the covariance matrix, the second eigenvalue, λ_2 of the PCA transform of g is equal to Var_u. This allows us to invert Equation 7 to arrive at the mapping, $\mathcal{M}$:

$$\mathcal{M}(\lambda_2) = \sqrt{\frac{1}{4} - \lambda_2}\,. \tag{8}$$

B Timings for Figures 1, 6 and 8

	Standard PM	Photon Relaxation	Diffusion-Based PM	Our method	Progressive PM
M / Absrbd. M	20M / 460k	20M / 460k	20M / 460k	20M / 460k	8.9B / 200M
Photon cast t	15.1s	15.1s	15.1s	15.1s	1h 49m
Radiance K	15	15	500	15	Variable
Feature detect t	-	4.6s	1.7s	4.0s	-
Relaxation t	-	14.0s	-	18.9s	-
Render t	3.4s	3.4s	2m 02s	3.4s	-
Total t	18.5s	37.1s	2m 18s	41.4s	1h 49m

Table I: Render timings for the close-up of the Cognac scene (Figure 1) for the various demonstrated techniques. Image resolution is 600 x 600 with 4x supersampling.

	Standard PM	Photon Relaxation	Diffusion-Based PM	Our method	Progressive PM
M / Absrbd. M	20M / 863k	20M / 863k	20M / 863k	20M / 863k	4.6B / 200M
Photon cast t	12.3s	12.3s	12.3s	12.3s	47m 21s
Radiance K	15	15	500	15	Variable
Feature detect t	-	9.1s	3.3s	5.3s	-
Relaxation t	-	27.3s	-	28.4s	-
Render t	4.3s	4.3s	1m 29s	4.3s	-
Total t	14.8s	48.2s	1m 44s	50.3s	47m 21s

Table II: Render timings for the close-up of the Prism scene (Figure 6) for the various demonstrated techniques. Image resolution is 600 x 600 with 4x supersampling.

	Standard PM	Photon Relaxation	Diffusion-Based PM	Our method	Progressive PM
M / Absrbd. M	1M / 787k	1M / 787k	1M / 787k	1M / 787k	635M / 500M
Photon cast t	2.5s	2.5s	2.5s	2.5s	26m 28s
Radiance K	15	15	500	15	Variable
Feature detect t	-	8.4s	3.4s	4.2s	-
Relaxation t	-	25.6s	-	26.9s	-
Render t	3.5s	3.5s	1m 57s	3.5s	-
Total t	6.0s	40.0s	2m 02s	37.1s	26m 28s

Table III: Render timings for the close-up of the Halogen scene (Figure 8) for the various demonstrated techniques. Image resolution is 600 x 460 with 4x supersampling.

C Full Comparison Renders for Figures 1, 6 and 8

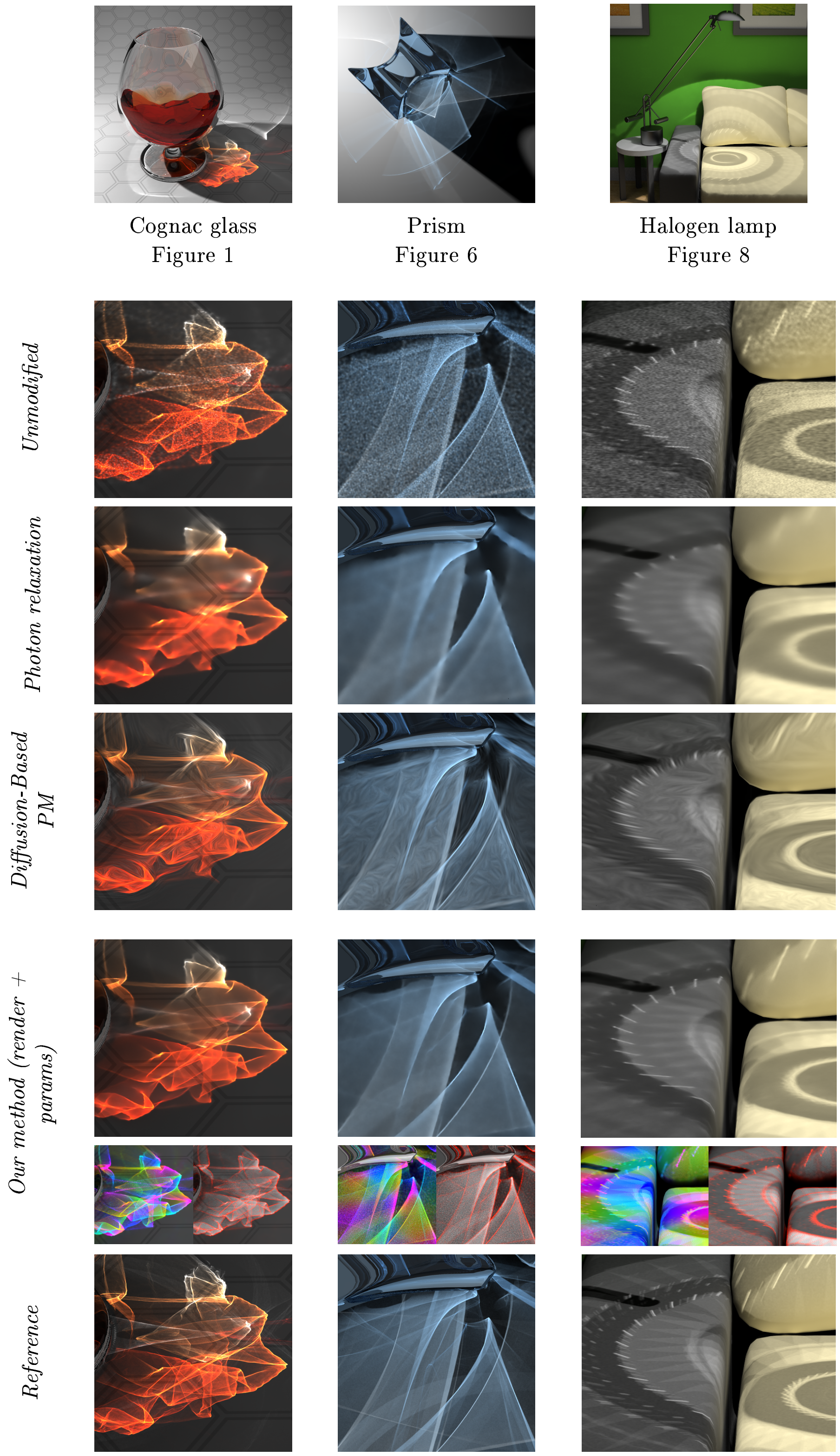

Figure 2: Side-by-side comparison for Figures 1, 6 and 8.
